# Measuring the impact of olive pomace enriched biscuits on the gut microbiota and its metabolic activity in mildly hypercholesterolaemic subjects

**DOI:** 10.1007/s00394-017-1572-2

**Published:** 2017-11-09

**Authors:** Lorenza Conterno, Francesca Martinelli, Matteo Tamburini, Francesca Fava, Andrea Mancini, Maddalena Sordo, Massimo Pindo, Stefan Martens, Domenico Masuero, Urska Vrhovsek, Claudia Dal Lago, Gabriele Ferrario, Mario Morandini, Kieran Tuohy

**Affiliations:** 1 OlioCRU s.r.l. Research and Development Group, Via Aldo Moro 1, 38062 Arco, TN Italy; 2 Casa di Cura Eremo di Arco s.r.l., Via XXI Aprile 1, 38062 Arco, TN Italy; 3 Department of Food Quality and Nutrition, Research and Innovation Centre-Fondazione Edmund Mach, San Michele all’Adige, Italy; 4 Genomics and Advanced Biology Unit, Research and Innovation Centre-Fondazione Edmund Mach, San Michele all’Adige, Italy

**Keywords:** Olive product, Prebiotic, Polyphenols, Metabolomic, Tyrosol glucoside, Tyrosol group

## Abstract

**Purpose:**

Olive pomace is a major waste product of olive oil production but remains rich in polyphenols and fibres. We measured the potential of an olive pomace-enriched biscuit formulation delivering 17.1 ± 4.01 mg/100 g of hydroxytyrosol and its derivatives, to modulate the composition and metabolic activity of the human gut microbiota.

**Methods:**

In a double-blind, controlled parallel dietary intervention 62 otherwise healthy hypercholesterolemic (total plasma cholesterol 180–240 mg/dl) subjects were randomly assigned to eat 90 g of olive pomace-enriched biscuit (olive-enriched product, OEP) or an isoenergetic control (CTRL) for 8 weeks. Fasted blood samples, 24-h urine and faecal samples were collected before and after dietary intervention for measurement of microbiota, metabolites and clinical parameters.

**Results:**

Consumption of OEP biscuits did not impact on the diversity of the faecal microbiota and there was no statistically significant effect on CVD markers. A trend towards reduced oxidized LDL cholesterol following OEP ingestion was observed. At the genus level lactobacilli and *Ruminococcus* were reduced in OEP compared to CTRL biscuits. A trend towards increased bifidobacteria abundance was observed after OEP ingestion in 16S rRNA profiles, by fluorescent in situ hybridization and by qPCR. Targeted LC–MS revealed significant increases phenolic acid concentrations in 24-h urine following OEP ingestion and 3,4-dihydroxyphenylacetic acid (DOPAC) and homovanillic acid, derivatives of hydroxytyrosol, were elevated in blood. A sex effect was apparent in urine small phenolic acid concentrations, and this sex effect was mirrored by statistically significant differences in relative abundances of faecal bacteria between men and women.

**Conclusion:**

Ingestion of OEP biscuits led to a significant increase in the metabolic output of the gut microbiota with an apparent sex effect possibly linked to differences in microbiota makeup. Increased levels of homovanillic acid and DOPAC, thought to be involved in reducing oxidative LDL cholesterol, were observed upon OEP ingestion. However, OEP did not induce statistically significant changes in either ox-LDL or urinary isoprostane in this study.

## Introduction

Olives and olive oil are important and characteristic components of the Mediterranean diet, a dietary pattern shown to improve on both physical and mental quality of life, and reduce the risk of chronic diet-associated disease, especially cardiovascular disease (CVD) [1]. Indeed, extra-virgin olive oil, as part of a Mediterranean style diet significantly reduced both the incidence of composite CVD end points and total mortality in the PREDIMED study [2]. Polyphenols, complex aromatic plant secondary metabolites, are independently linked to these health effects [1, 3]. Olives and various olive oils and extracts have been shown to mediate different health effects in humans, many associated with CVD risk. Olive extracts have been reported to lower systolic blood pressure (SBP) and diastolic blood pressure (DBP) from baseline in both hypertensive and pre-hypertensive individuals [4–7] and to improve plasma lipid profiles in both normo-lipidaemic and hypercholesterolaemic subjects [4, 6, 8–10]. Olive extracts have also been found to induce acute reductions in arterial stiffness [11], which agrees with data suggesting that olive oil and olive extract significantly improve vascular function [12, 13] and blood pressure [14]. Currently, these improvements are thought to be associated with polyphenol-rich olive oil fractions rather than other bioactives which may be present [15, 16]. In contrast, other studies have failed to demonstrate significant modulation of CVD biomarkers upon olive extract ingestion. For example, olive leaf extracts did not appear to improve plasma lipids [7, 17], ambulatory blood pressure, cytokines and/or carotid intima-media thickness [17] in different studies. However, the European Food Safety Authority (EFSA) has recognized a specific health claim for the polyphenol extract of olive for protection of LDL cholesterol particles against oxidative damage—although they also noted that there is a lack of evidence for other health claims including maintenance of normal blood pressure and HDL cholesterol levels, reduced inflammation and improved gastrointestinal function [18]. EFSA considers that the claim that “consumption of olive oil polyphenols contributes to the protection of blood lipids from oxidative damage” reflects the scientific evidence, and that a dose of 5 mg of hydroxytyrosol and its derivatives (e.g. oleuropein complex and tyrosol) in olive oil should be consumed daily for food products to bear the health claim. In olive, the majority of polyphenols present belong to the tyrosol group [hydroxytyrosol (HT), tyrosol (TYR) and conjugated forms like oleuropein]. These conjugated forms are extensively hydrolyzed in the stomach [19] to HT and TRY, which are either absorbed in the small intestine and undergo extensive phase I and II biotransformation or reach the colon where they undergo biotransformation by the resident microbiota [19, 20]. The most common derivatives are small phenolic acids like homovanillic acid (HVA), dihydroxyphenylacetic acids (DHPAA), hydroxyphenylacetic acid (HPAA), protocatechuic acid and benzoic acids for example. The impact of olives or their constituent parts on the composition and metabolic activity of the human intestinal microbiota is, however, poorly understood. One recent study has shown that thyme phenolic compounds at different doses in olive oil can induce a small increase in bifidobacteria using the quantitative culture-independent method fluorescent in situ hybridization (FISH), with the suggestion that this change in microbiota could be related to improved LDL cholesterol oxidative status [21]. Similarly, a sex effect in terms of metabolism of HT and related compounds has been observed in rats with the suggestion that differential excretion of HT derivatives between male and female animals might be due to sex-linked differences in enterohepatic circulation [22]. However, no data are reported for differences in metabolism of these compounds between men and women or indeed, whether such differences if they do exist, could be due to sex-specific differences in gut microbiota. To date, no studies have reported whether olives or olive pomace can impact on the relative abundances of the human gut microbiota, on the diversity of the gut microbiota and only a few studies have specifically addressed the metabolic end products produced by combined host–microbiota co-metabolism of olive polyphenols [20, 21].

Olives contain many potential biologically active compounds such as polyphenols, dietary fibre (including pectin), oleic acid, linoleic acid and other beneficial fats, tocopherols, phytosterols and squalene. Olive oil is extracted from the fruit of *Olea europaea*, leaving waste in the form of olive water and solid olive pomace. The olive pomace and wastewater produced from oil extraction processes contain macromolecules such as polysaccharides, lipids, proteins and polyphenolic compounds (mainly of the tyrosol group) which can range from 1 to 8 g/l [23] in wastewater and 2.9 to 3.7 mg/l in olive pomace [24]. The annual worldwide production of olive oil is about 2 million metric tons reported for 2015/16 (average: COI, 2017). For each ton of olive oil the waste produced is dependent on the fruit quality, ripeness and extraction technology and typically ranges from 2.75 to 4 tons of olive pomace and 1–8 m^3^ of wastewater [25–27]. In addition these olive mill wastes are produced in significantly large quantities during the short olive production season and because the waste cannot be disposed of through ordinary waste treatment systems, disposal of wastewater and olive pomace is a major environmental problem and cost to the industry. Given the push towards a green economy, there is a stimulus for the oil industry to move towards a circular economic model, reducing waste production or adding value to waste streams, developing added value secondary product lines. To this end, we have developed an olive pomace extract as a functional food ingredient. This olive pomace, which delivers 17.1 ± 4.01 mg/100 g HT and its derivatives, is also rich in fibres and our preliminary in vitro data showed it was fermentable and leads to increased numbers of bifidobacteria in pH-controlled faecal batch cultures (data not shown). For this current study, we have incorporated this olive pomace into a biscuit formulation. For flavour, texture and constitutional reasons, other ingredients were also necessary including extra virgin olive oil and flours from chestnut, pea and buckwheat, but the olive pomace remained the dominant ingredient and the dominant source of polyphenols (> 90% of polyphenols present in the final biscuit). Here we report the effect of this olive pomace-enriched biscuit on the human gut microbiota, their metabolic output and on various biomarkers of CVD and inflammation. The study was conducted jointly by researchers at Fondazione Edmund Mach (San Michele all’Adige, Italy), OlioCru s.r.l (Arco, Italy) and the study centre was the Casa di Cura Eremo di Arco s.r.l. (Italy), a private specialist clinic specialized in treating CVD.

## Materials and methods

### Study design

**Fig. 1:**
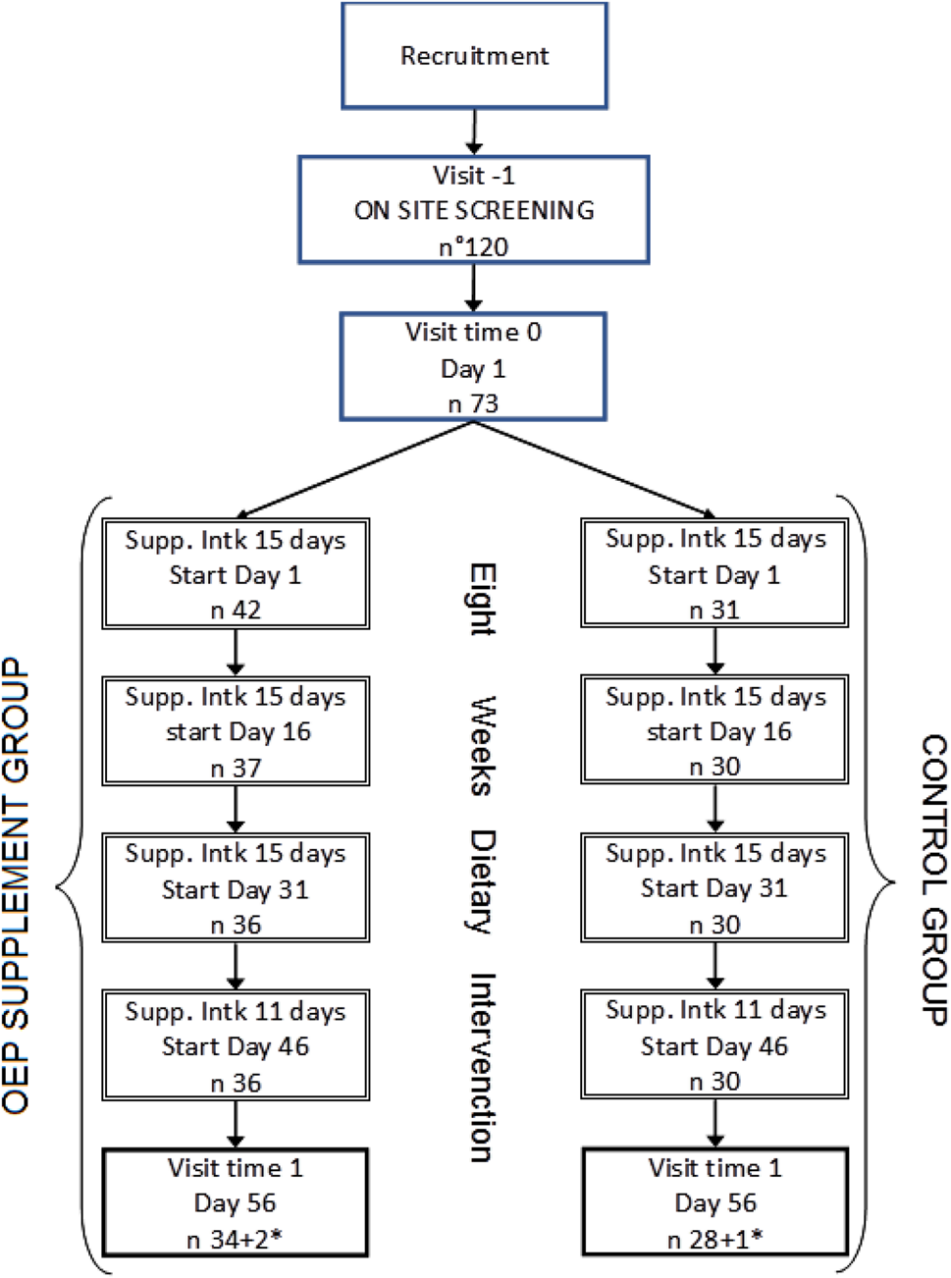
Schematic representation of study design. *At the end of the study because of antibiotic usage or non-compliance for consumption of the product, two people were excluded from the olive-enriched product (OEP) group and one from the control group

The study was a double-blind, randomized, controlled, parallel trial (Italy protocol Prebioil2 number: 6/2015, Clinicaltrials.gov ID: NCT02664428, see Fig. 1 for the study design). This study was conducted according to the guidelines laid down in the Declaration of Helsinki, and all procedures involving human subjects were approved by the Ethic Committee for Clinical Trials of the Trento Azienda Provinciale Servizi Sanitari (APSS). The clinical trial was carried out at the Casa di Cura Eremo di Arco (Arco, TN, Italy) between November 2015 and June 2016. The primary outcome measure was the faecal microbiota analysis by Illumina sequencing and fluorescence in situ hybridization (FISH) carried out with probe for *Bifidobacterium* spp., *Lactobacillus* spp. and *Ruminococcus* spp. The secondary outcome measures were related to cholesterol analysis in plasma [total, LDL, HDL cholesterol, oxidized LDL, triglycerides, apolipoproteins A–I and B (APO A and APO B)] together with the analysis of the variation of polyphenols and their metabolites in plasma and urine. Additional measures were the analyses of the anthropometric indices, the fasting plasma insulin, glucose and C-reactive protein (CRP) and the analysis of isoprostane F2 in urine. This study was powered for changes in blood LDL cholesterol and changes in faecal bifidobacteria. Since previous studies have shown that fewer individuals are required for measuring changes in faecal bifidobacteria and because of its clinical significance, the sample size calculation was performed only for changes in LDL cholesterol levels. Based on measures from a previous parallel trial design using similar products [28], we assessed as clinical significant end point the average LDL decrease of 15.44 mg/dl with a standard deviation of 14.28 mg/dl. According to Snedecor and Cochran equation with significance value *α* equal to 0.05 and a 90% power (1 − *β*) [29], the minimum number of subjects to enrol was 28. Taking into account possible dropouts, a total of 73 subjects were enrolled and randomly assigned to the dietary intervention with the product under investigation (OEP) or to the control dietary intervention.

### Intervention foods

The study product “PreBiÒ^®^”, herein called olive pomace-enriched product (OEP), was a bakery product comprising dehydrated food-grade olive powder, together with chestnut, peas, buckweat flour, extra virgin olive oil (EVOO), salt and sugar according to the OlioCRU proprietary recipe, and the OlioCRU pending Patent Process. The product was prepared at a local bakery. The product contained about 411 ± 25 mg/100 g of total biophenols measured according to the COI method of which 17.1 ± 4.01 mg/100 g belong to the tyrosol group polyphenols and were measured according to Gasperotti et al. [30].

The product corresponded to the energy intake of 434 Kcal/100 g (average of three replicates).

Subjects were provided with single 90 g daily doses and instructed to consume one each day of the 8 weeks of intervention. The control product comprised wheat flour, sugar, salt and low-polyphenol EVOO, food colourings in safety-approved quantities to match the test product OEP as closely as possible in appearance, taste, texture. The product contains less than 1 mg/100 g of total biophenols measured according to the IOC official method (IOC 2009) of which 0.7 ± 0.5 mg/100 g belong to the tyrosol group. The product corresponds to an energy intake of 419 Kcal/100 g (average of three replicates). Table 1 shows the gross nutritional composition of the OEP and control (CTRL) biscuits.

**Table 1:** Food supplements’ nutritional value (g/100 g, mean ± standard deviation) (**a**) and polyphenol composition (μg/g) (**b**)

	OPE (g/100 g)	Ctrl (g/100 g)
A		
Carbohydrates	49.2 (± 1.04)	70.9 (± 2.19)
Lipids	18.5 (± 0.40)	8.1 (± 2.40)
Proteins	16.1 (± 4.39)	10.9 (± 0.92)
Dietary fibre	13.3 (± 0.17)	3.4 (± 0.35)
Salt	0.7 (± 0.2)	0.7 (± 0.2)

Compound	OEP		Ctrl	
	Mean	SD	Mean	SD
B				
Cinnamic acid	0.39	0.04	< 0.005	–
Vanillin	0.26	0.02	< 0.01	–
Esculin	0.14	0.01	< 0.005	–
Neochlorogenic acid	0.40	0.18	< 0.01	–
Chlorogenic acid	55.08	4.24	< 0.025	–
Fertaric acid	0.09	0.02	< 0.01	–
*t*-Coutaric acid	0.68	0.13	< 0.01	–
Apigenin	3.30	0.13	< 0.0025	–
Luteolin	10.14	3.40	1.19	1.26
Luteolin-7-*O*-Glc	8.95	0.88	< 0.0025	–
Apigenin-7-Glc	1.23	0.07	< 0.0025	–
Naringenin	0.17	0.03	< 0.0025	–
Epicatechin	0.48	0.36	< 0.1	–
Kaempferol	3.44	0.52	< 0.0025	–
Quercetin	2.57	0.26	< 0.0025	–
Quercetin-3-Rha	1.52	0.11	< 0.005	–
Kaempferol-3-Glc	2.25	0.39	< 0.0025	–
Syringetin-3-Glc + syringetin-3-Gal (as syr-3-glc)	0.14	0.02	< 0.0025	–
Kaempferol-3-rutinoside	0.07	0.02	< 0.0025	–
Arbutin	0.21	0.05	0.31	0.11
4-Hydroxybenzoic acid	1.02	0.33	0.81	0.12
*p*-Coumaric acid	1.01	0.38	0.71	0.09
Vanillic acid	2.98	0.43	1.37	0.18
Caffeic acid	1.19	0.11	< 0.025	–
Isoferulic acid	0.74	0.03	1.04	0.18
*t*-Ferulic acid	21.21	0.03	33.65	4.75
Ellagic acid	6.40	1.26	< 0.025	–
Pyrocatechol	7.20	0.60	9.09	2.36
Protocatechuic acid	4.69	0.65	< 0.025	–
Scopoletin	0.26	0.08	< 0.0025	–
Cryptochlorogenic acid	3.65	0.64	< 0.025	–
Quercetin-3-Glc + quercetin-3-gal(as que-3-glc)	0.60	0.37	< 0.005	–
Rutin	2.32	0.42	< 0.0025	–
Hydroxytyrosol	8.11	4.41	0.79	0.32
Chrysoeriol	0.13	0.02	0.07	0.02
Salidroside	121.19	34.59	< 0.25	–
Pinoresinol	1.11	0.15	< 0.0025	–
Oleuropein	5.72	0.78	< 0.005	–
Diosmetin	0.13	0.03	0.06	0.01
Tyrosol	8.86	0.86	6.31	0.72

Values below the limit of quantification are not shown

### Recruiting

Healthy volunteer were recruited through advertisement in the geographical area of Arco (TN) in the northeast of Italy. Advertisement was carried out via flyer, posters and e-mails. Individuals who answered the call were asked their weight and height and those who corresponded to BMI in the range established by the inclusion criteria were given an appointment for the general health assessment to determine conformity with other inclusion/exclusion criteria. At the clinic a health and lifestyle questionnaire was completed, eco-cardiogram and physical examinations were performed together with collection of urine and fasted blood samples. The inclusion criteria were non-smoking status, age between 30 and 65 years, BMI between 20 and 29.9 kg/m^2^, plasmatic total cholesterol between 180 and 240 mg/dl, being free from chronic disease, including cardiovascular disease, diabetes, cancer, inflammatory or digestive disorders. Pregnancy or breastfeeding, and individuals consuming more than 21 U/week of alcohol were excluded. Subjects were excluded if taking statins or other medication or dietary supplements that may affect lipids. Uncontrolled hypertension was an exclusion criteria, and to be included the subjects were either not hypertensive or under hypertensive medication and presenting with average SBP below 121 mmHg and average DBP 90 mmHg. Subjects with food allergies or intolerances were also excluded.

### Randomisation and blinding

Treatment allocation was done using a random block design. A six-digit code was assigned to each recruited individual. The products was randomly assigned to the subject code by an external individual by picking codes form a bag in a blind manner but matching groups by age and sex. Each daily dose was prepared in a food-grade sealed box enveloped in dark green paper sachet. The treatment codes were kept offsite and not released until statistical analysis was complete. Therefore, allocation concealment was achieved and both researchers and subjects were blinded to which product was being consumed at which time.

### Screening

Clinical visits took place at the beginning of week 1, and at the end of week 8 at the Casa di Cura Eremo di Arco s.r.l., Via XXI Aprile 1, 38062 Arco (TN) Italy. Subjects arrived for screening fasted and measurements of height and weight were taken in a Kern scale (Kern & Sohn, Balingen, Germany) with stand and height rod (Mod MPB300K100P) to calculate BMI. Blood pressure was measured after 5-min rest, seated and with the subject’s dominant arm resting on a table, using an Omron digital blood pressure equipment (HEM-705 CP).

Three readings were taken 60 s apart and averaged. Subjects were not permitted to talk during measurements. Blood pressure was measured at the screening visit (T-1) and later at the beginning (T0) and at the end of the treatment (T1). Blood samples obtained via single venepuncture were collected into heparin and EDTA vacutainers (BD) and used for the analysis established for health. A total of 73 suitable subjects were identified and accepted onto the trial.

Each subject was informed about the study aims and procedure to allow them to sign and informed consent. Eligible participants were asked to provide written informed consent to take part in the study.

### Compliance measures

Subjects were asked to return all remaining full or empty daily packages of test product after 8 week intervention. Remaining material were weighed and recorded. Subjects were asked to complete weekly online questionnaires and supplied with daily tick sheets.

### Faecal sample collection

Volunteers were provided with a sealable pot, and sterile bag to collect the stool sample, each of them were instructed to collect the stool sample in the sterile bag, put it into the pot and, prior to sealing the pot, to add an atmosphere generation system AnaeroGen Compact (Thermo Scinetific). This ensured an anaerobic environment during sample transport. Samples were collected and treated for further analysis or stored at − 80 °C within 24 h.

### Urine sample collection

Volunteers were supplied 3-l sterile containers to collect the 24-h urine samples. Each container was added with 15 ml of 3M hydrochloridric acids as a preservative. After collection, the total urine volume was measured and the samples for further analysis were prepared and stored at − 80 °C.

### Biochemical measures

Blood collected in EDTA and heparin vacutainers was centrifuged at 1550×*g* for 15 min to separate plasma. Plasma was immediately analysed or stored in low-binding Eppendorf tubes (Axygen, Tewksbury MA, USA) at − 80 °C until analysis. Total cholesterol (TC), HDL cholesterol (HDL), LDL cholesterol (LDL), apolipoprotein A1 (Apo A1) and B (Apo B) triglycerides (TG), CRP, glucose (GLU) and insulin (Ins) were measured at Clinic Casa di Cura Eremo Laboratory (Arco, TN, Italy), using ILab 650 chemistry analyser (Instrumentation Laboratories UK Ltd, Warrington, United Kingdom) for all measures except insulin which was measured using a Roche COBAS 6000.

Oxidized LDL was measured in duplicate via an ELISA kit (Mercodia, Sweden).

F2 isoprostane urinary total (conjugated and non-conjugated) were measured using the enzyme immunoassay based kit Urinary Isoprostane Elisa Kit (Oxford Biomedical Research, USA).

### Metagenomic analysis

A whole fresh stool sample was collected at T0 and T1. The stool was stored at − 80 °C. DNA was extracted from 100 mg stool aliquots using Power faecal DNA extraction kit (MOBio), following the manufacturer’s instructions. Metagenomic sequencing was performed to evaluate microbiota diversity and genus-level abundances. Using the specific bacterial primer set 341F (5′ CCTACGGGNGGCWGCAG 3′) and 806R (5′GACTACNVGGGTWTCTAATCC 3′) with overhang Illumina adapters, total genomic DNA was subjected to PCR amplification by targeting a ~ 460-bp fragment of the 16S rRNA variable region V3–V4. PCR amplification of each sample was carried out using 25-μl reactions with 1 μM of each primer. Specifically 12.5 μl of 2× KAPA HiFi HotStart ReadyMix, 5 μl forward primer, 5 μl reverse primer were used in combination with 2.5 μl of template DNA (5 ng/μl). All PCR amplifications were carried out using a GeneAmp PCR System 9700 (Thermo Fisher Scientific) and the following steps—melting step; 94 °C for 5 min (one cycle), annealing step; 95 °C for 30 s, 55 °C for 30 s, 72 °C for 30 s (30 cycles), extension step; 72 °C for 5 min (1 cycle). The PCR products were checked on 1.5% agarose gel and cleaned from free primers and primer dimer using the Agencourt AMPure XP system (Beckman Coulter, Brea, CA, USA) following the manufacturer’s instructions. Subsequently dual indices and Illumina sequencing adapters Nextera XT Index Primer (Illumina) were attached by seven-cycle PCR (16S Metagenomic Sequencing Library Preparation, Illumina). The final libraries, after purification by the Agencourt AMPure XP system (Beckman), were analysed on a Typestation 2200 platform (Agilent Technologies, Santa Clara, CA, USA) and quantified using the Quant-IT PicoGreen dsDNA assay kit (Thermo Fisher Scientific) by the Synergy 2 microplate reader (Biotek). Finally all the libraries were pooled in an equimolar way in a final amplicon library and analysed on a Typestation 2200 platform (Agilent Technologies, Santa Clara, CA, USA). Bar-coded library were sequenced on an Illumina® MiSeq (PE300) platform (MiSeq Control Software 2.0.5 and Real-Time Analysis software 1.16.18). Differences in relative abundance after intervention (V2–V1) with treatment W or P were analysed using non-parametric *t* test (Mann–Whitney *U* test).

### Quantitative microbial molecular techniques

#### Flow cytometry (FCM) fluorescent in situ hybridization (FISH)

1:10 dilution (wt:vol) of the faecal sample was prepared by weighting out 2–3 g of faecal sample and diluting it with 1M PBS on the scale (e.g. 3 g of sample + 27 g of PBS, considering that 1 ml of 1M PBS weights 1 g). The diluted sample was homogenized using a Stomacher 400 (Seward) at the speed of 230 rpm for 2 min or until it appeared homogeneous. Ten millilitre of the suspension was transferred into a 15-ml falcon tube containing glass beads; the tube was mixed by vortexing for about 30 s, then centrifuged at 1100 rpm for 2 min to pellet fibrous particles. Then 375 μl of the suspension were transferred into a 1.5-ml Eppendorf tube containing 1125 μl of 4% paraformaldehyde (PFA). The suspension was fixed in 4% PFA for 4–24 h at 4 °C. After fixation the tubes were centrifuged at 13,000 rpm for 5 min and the pellet were resuspended in 1 ml of filter-sterilized 1M PBS. This washing procedure was repeated twice, then the tubes were centrifuged again at 13,000 rpm for 5 min, supernatant was carefully removed and the pellet was finally resuspended in 150 μl of filter-sterilized 1M PBS. One hundred and fifty (150) microlitre of absolute ethanol was added and the samples were mixed by inversion and immediately stored at − 20 °C. For FCM-FISH analysis, 10 μl of the fixed faecal sample was resuspended in 190 μl of PBS 1X sterile. Every step was done in 96-well plates. After resuspending, the sample was centrifuged at 4000 rpm for 15 min. The sample was resuspended in 200 μl of Tris–EDTA buffer and then centrifuged another time at 4000 rpm for 15 min. For hybridisation with the probes specific for *Lactobacillus*/*Enterococcus* spp. [31], *Bifidobacterium* spp. [32] and *Ruminococcus* spp. [33] that needed lysozyme treatment to render the cell wall more permeable to the probes, we resuspended the sample in 200 μl of Tris–EDTA containing 1 mg/ml of lysozyme and incubated for 10 min at room temperature. After that period, sample was washed with centrifugation and resuspended in 50 μl of hybridization buffer [0.9 M NaCl, 20 mM Tris–HCl (pH 7.5), 0.1% [wt/vol] sodium dodecyl sulphate (SDS)] with 5 μl of 50 ng/μl fluorescently Cy5-labelled oligonucleotide probe and incubated at the appropriate hybridization temperature (Table 2). Washing was repeated and the sample was resuspended in 200 μl of hybridization buffer without SDS and incubated at the appropriate wash temperature (Table 2). After washing, sample was resuspended in SYBR Green, used to enumerate the total cells. SYBR Green binds to DNA and the resulting complex absorbs blue light (*λ*_max_ = 497 nm) and emits green light (*λ*_max_ = 520 nm). A blank sample (without the fluorescently Cy5-labelled oligonucleotide probe and without the SYBR Green) was prepared and for every sample, following the same steps as per the hybridized sample, to set the threshold of the gates of the flow cytometer that permits the revelation of the microbial species and exclude the false positives due to the potential autofluorescence of the sample. FCM was performed using Guava easyCyte™ Single Sample Flow Cytometer (Millipore) with a single blue (488 nm), dual blue and red (642 nm), or triple blue, red, and violet (405 nm) excitation lasers that provided 12 simultaneous detection parameters, including 10 fluorescent colours plus forward and side scatter for size and granularity determination. The FCM parameters were adjusted to give particle counts of 1000 events in total. Data were analysed by the InCyte software, version 4.1.1. To avoid loss of the signal intensity of hybridized cells, the samples were kept in the dark until the FCM analysis. Results were expressed as the percentage of cells hybridized with the group-specific-Cy5 probe calculated on the total bacteria, counted after SYBR Green staining. Also absolute numbers were obtained.

**Table 2:** Anthropometric and clinical parameters, mean (± standard deviation), before (T0) and after (T1) dietary intervention with olive-enriched product (OEP) or control (Ctrl)

	Dietary supplement		Dietary supplement		ANOVA *p* value
	OEP		Ctrl		
	T0	T1	T0	T1	
SYS (mmHg)	120 (± 11.4)	122 (± 12.4)	122 (± 15.0)	121 (± 17.2)	0.416
DIA (mmHg)	78 (± 7.4)	78 (± 7.7)	78 (± 9.7)	77 (± 9.8)	0.586
WM (cm)	84 (± 11.9)	84 (± 11.)	83 (± 11.7)	82 (± 10.9)	0.931
HM (cm)	102 (± 6.4)	102 (± 6.3)	102 (± 5.5)	102 (± 5.9)	0.944
Weight (kg)	70 (± 12.1)	70 (± 12.1)	71 (± 11.6)	70 (± 11.6)	0.974
BMI (kg/m^2^)	24 (± 3.4)	24 (± 3.5)	24 (± 3.0)	24 (± 3.0)	0.956
TC (mg/dl)	204 (± 22.0)	202 (± 20.7)	217 (± 22.0)	213 (± 26.9)	0271
HDL (mg/dl)	64 (± 11.7)	64 (± 11.5)	66 (± 14.0)	65 (± 12.8)	0.395
TC/HDL	3.3 (± 0.7)	3.2 (± 0.7)	3.5 (± 0.8)	3.4 (± 0.9)	0.592
LDL (mg/dl)	120 (± 18.6)	118 (± 20.4)	129 (± 21.7)	126 (± 24.7)	0.378
TG (mg/dl)	92 (± 32.9)	94 (± 36.1)	96 (± 51.3)	95 (± 49.0)	0.924
Apo A1 (mg/dl)	144 (± 15.9)	145 (± 16.5)	145 (± 21.3)	144 (± 17.0)	0.636
Apo B (mg/dl)	91 (± 13.8)	91 (± 15.3)	95 (± 14.6)	95 (± 17.7)	0.920
Glu (mg/dl)	93 (± 7.0)	93 (± 6.9)	93 (± 10.9)	95 (± 10.8)	0.418
Ins (mcU/ml)	7.0 (± 5.3)	6.1 (± 2.8)	6.7 (± 5.1)	6.5 (± 5.0)	0.500
CRP (mg/L)	1.5 (± 2.7)	1.4 (± 2.2)	1.1 (± 1.0)	1.2 (± 1.3)	0.964
Ox LDL (U/L)	59.8 (± 16.7)	57.3 (± 17.2)	63.5 (± 17.2)	64.3 (± 21.3)	0.634
F2 Iso (μg/24 h)	2.20 (± 0.90)	2.22 (± 0.84)	2.04 (± 0.68)	2.01 (± 0.94)	0.739

*p* value is relative to one-way ANOVA on the difference between T0 and T1

*SYS* systolic pressure, *DIA* diastolic pressure, *WM* waist measure, *HM* hips measure, *BMI* body mass index, *TC* plasma total cholesterol, *HDL* plasma high-density lipoproteins, *LDL* plasma low-density lipoproteins, *TG* plasma triglycerides, *Apo* Apolipoproteins in plasma, *Glu* plasma glucose, *Ins* plasma insulin, *CRP* plasma reactive C protein, *oxLDL* plasma oxidized LDL, *F2 Isp* total 24-h urine Isoprostane F2

#### Quantitative PCR

DNA extraction was performed using the FastDNA™ SPIN Kit for Feces (MP Biomedicals). Amplifications were performed with sets of primers specific for *Bifidobacterium* spp. [Bif F: TCG CGT C(C/T)G GTG TGA AAG; Bif R: CCA CAT CCA GC(A/G) TCC AC] and for total bacteria (Bact 1369: CGG TGA ATA CGT TCC CGG; Prok 1492 TAC GGC TAC CTT GTT ACG ACTT). Reactions were performed at the specified conditions (see reference) using SsoFAST Evagreen SupemixKit (BIO RAD) and a Lightcycler 480 PCR machine (Roche). Quantifications were done using standard curves obtained by amplifying pure cultures of Bb12 which had been previously quantified by plate counting. For total bacteria a mixture of bacterial DNA was obtained by pooling the total faecal genomic DNA from four faecal samples, which had been previously enumerated using FCM-FISH.

### Metabolite analysis

Targeted metabolomics analysis by UHPLC–ESI-MS/MS was carried out as previously described [30, 34] on 24-h urine and fasted blood samples. After methanol extraction of polyphenols, analysis was performed by an ultra-performance liquid chromatographic system (UHPLC) coupled with a tandem mass spectrometer. The system used was an ACQUITY UPLC system coupled to a Xevo TQ triple quadrupole via an electrospray (ESI) interface (Waters, Milford, MA, USA). The separation was performed with a Waters ACQUITY UPLC column HSS T3 (100 mm × 2.1 mm, 1.8 μm) equipped with a guard column. The injection volume was 5 μl. Mobile phases of 0.1% formic acid in Milli-Q water (A) and 0.1% formic acid in acetonitrile (B) were used. Chromatographic separation was performer using a gradient as follows: 0 min, 5% B; 0–3 min, 5–20% B; 3–4.30 min, 20% B; 4.30–9 min, 20–45% B; 9–11 min, 45–100% B; 11–14 min, 100% B; and 14.01–17 min, 5% B as equilibration time. For calibration, a standard mixture of polyphenol metabolites was serially diluted in aqueous methanol (50:50) at a concentration range of 0.01–20 mg/l. Quantitative data were processed withTargetlynx software (Masslynx, Waters).

### Statistical analysis

Statistical analysis was performed using STATISTICA 13.1 statistics software for data analysis. Data were checked for normality using the Kolmogorov–Smirnov and Shapiro–Wilk tests. Treatment effects were assessed using one-way analysis of variance, or non-parametric Mann–Whitney test. Treatments were compared to each other using a paired Student’s *t* test. *p* values < 0.05 were deemed statistically significant. qPCR data analysis was performed using factorial ANOVA (factors: treatment, time) with FDR correction. Urine and plasma metabolite data analysis was performed using factorial ANOVA (factors: treatment, time) with FDR correction.

## Results

### Study report

A total of 73 suitable subjects were identified and were accepted onto the trial to begin the dietary intervention. Eight did not finish the study. Two subjects dropped out because of illness not related to the intervention (flue and surgery), one for family-related issues, five did not like the taste of the product and dropped out. Exclusion occurred because of deviation from the protocol: one volunteer declared after finishing the study to have taken antibiotics and two subjects were excluded because they did not consume the product as directed (more than 25% returned unconsumed). In total 62 people completed the study successfully and were included in the statistical analysis. In detail, 32 females and 30 males, between 30 and 65 years old, with BMI from 20 to 29.9 (average 24 ± 3.4) and total cholesterol ranging between 180 and 240 mg/dl completed the study. Female group had an average age of 48 years (± 8.5), while the male group average age was 49 (± 9.6). Between the treatment groups at baseline, blood pressure (average 120/74 and 122/74), BMI (average 24 and 24) and total cholesterol (average 204 and 217), no significant differences were measured (Table 2).

### Faecal microbiota analysis

#### 16S rRNA gene community analysis

A total of 8,924,305 reads was generated, with an average of 68,648.5 ± 36,038.2, mean ± SD, high-quality 16S rRNA gene sequences per stool sample. Microbiota diversity was evaluated for alpha diversity (diversity within a sample, Chao index) and beta diversity (diversity between samples, Bray–Curtis dissimilarity) using QIIME (https://www.qiime.org, [35]). Sequences with expected error rate > 1.5% and length < 400 bp were removed from analysis. After filtering and chimera removal, 1418.2 ± 623.9, mean ± SD, Operational Taxonomic Units (OTU) were obtained on average per sample analysed. OTUs that were present in less than 25% of the samples were removed.

**Fig. 2:**
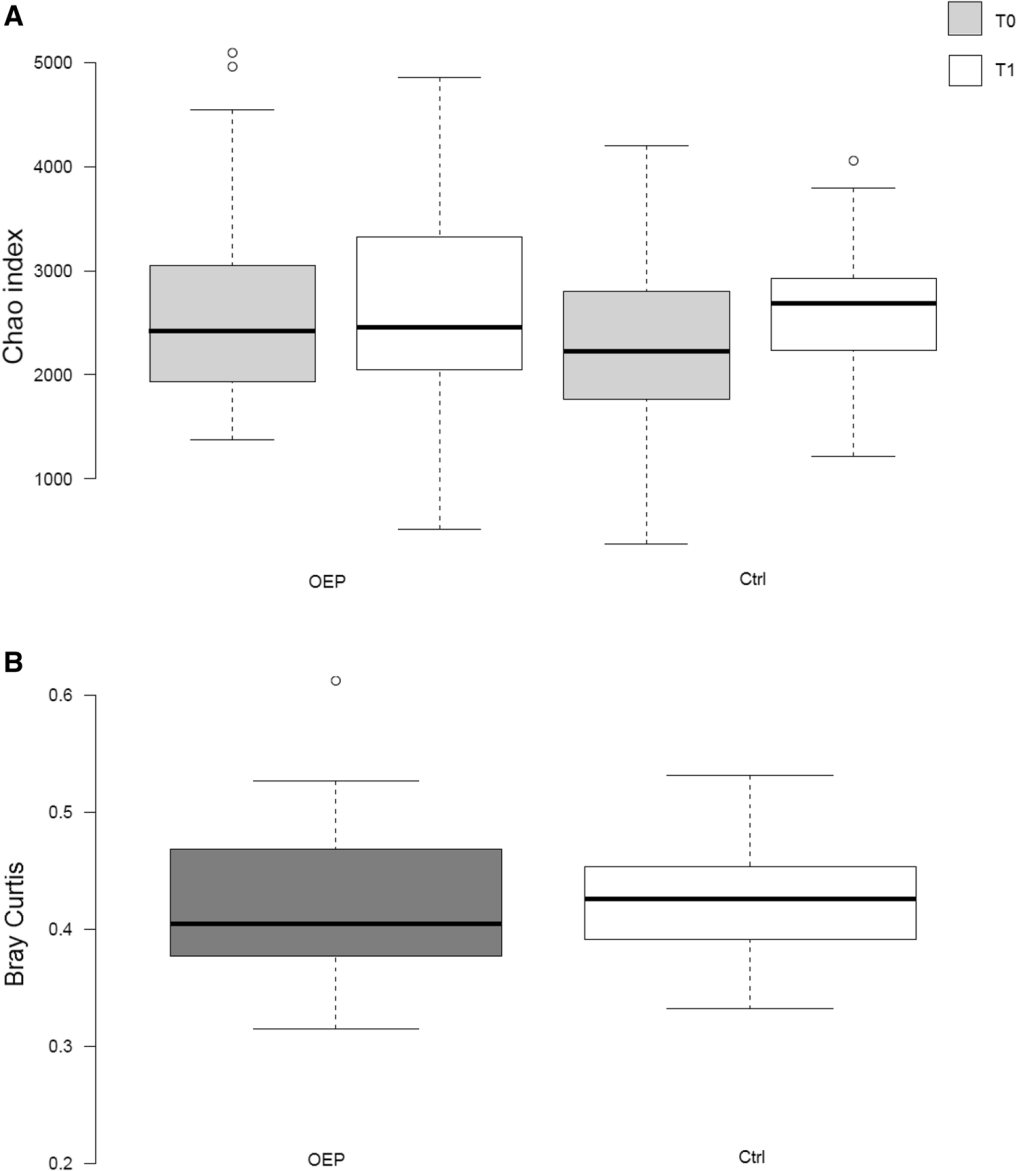
Alpha- (**a**) and beta-diversity (**b**) indexes after 16S rRNA metagenomic analysis of faecal samples collected before (T0) and after (T1) dietary intervention. Center lines show the medians; box limits indicate the 25th and 75th percentiles as determined by R software; whiskers extend 15 times the interquartile range from the 25th and 75th percentiles; outliers are represented by dots

The two intervention treatments did not show statistically significant differences in alpha diversity (2634.54 ± 943.69 vs 2682.012 ± 1177.8 and 2419.24 ± 1170.06 vs 2753.09 ± 875.9 Chao index, V1 vs V2, OEP and CTRL, respectively; *p* = 0.29) and beta diversity (0.433 ± 0.087 vs 0.43 ± 0.064 Bray–Curtis dissimilarity index, mean ± SD, OEP vs CTRL, respectively; *p* = 0.66) (Fig. 2a, b).

**Fig. 3:**
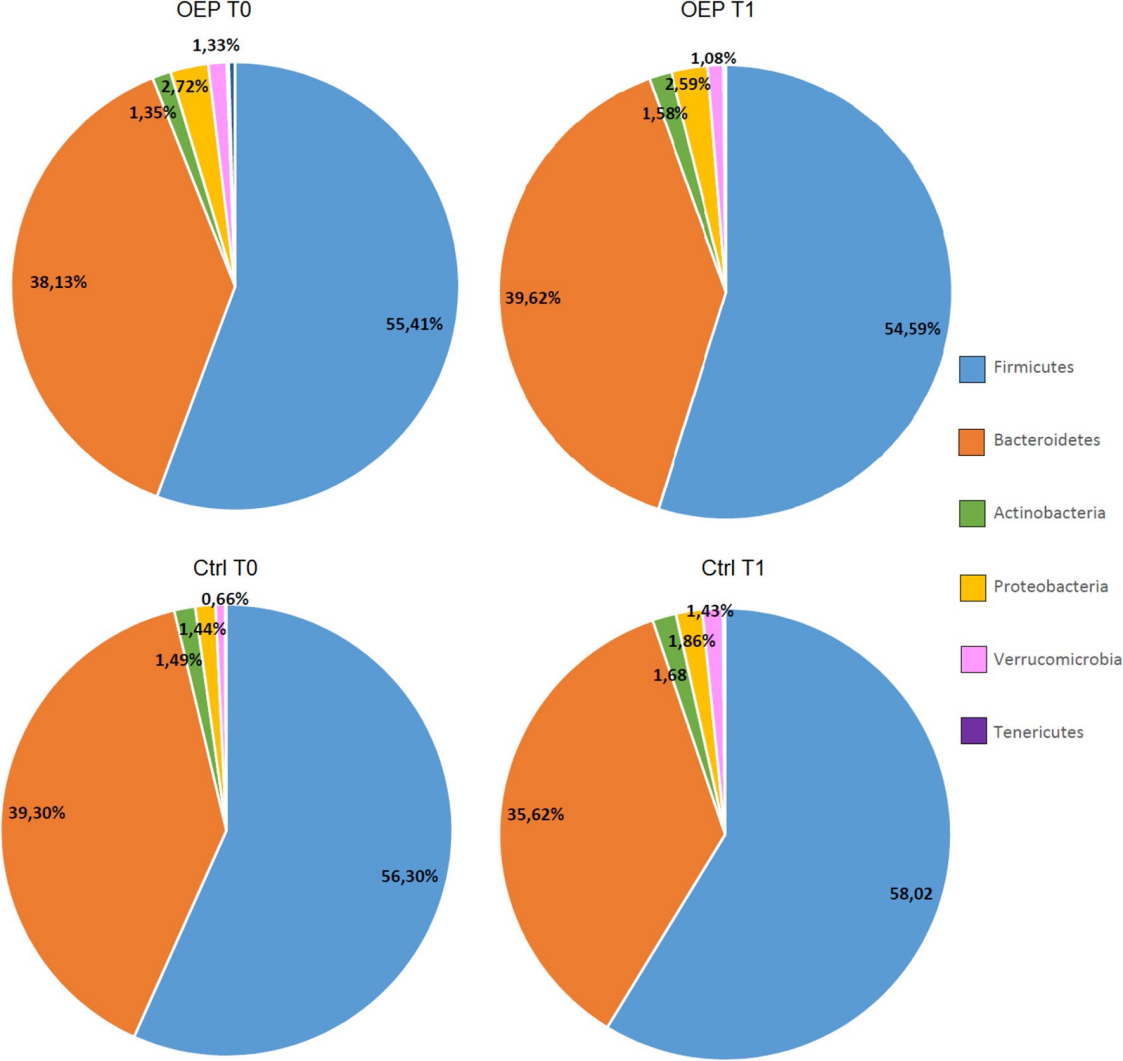
Percentage relative abundance after 16S rRNA metagenomics analysis of dominant bacterial phyla in faecal samples collected before (T0) and after (T1) dietary intervention with olive-enriched product (OEP) or control product (Ctrl)

At the phylum level, although there was a trend towards increased Actinobacteria with both treatments, there was no statistically significant change within or between treatments over the course of the trial (Fig. 3).

At a genus taxonomic level, a significant although very small increase of *Lactobacillus* and *Ruminococcus* was observed after intervention with CTRL compared to OEP (0.22 ± 1.21 and 0.27 ± 1.45 vs 0.07 ± 0.33 and 0.05 ± 0.31, *p* = 0.042 for *Lactobacillus*; 0.54 ± 0.60 and 0.70 ± 0.76 vs 0.77 ± 0.93 and 0.43 ± 0.38 for *Ruminococcus, p* = 0.0191, percentage relative abundance, V1 and V2, CTRL vs OEP, respectively). Also very small changes in relative abundance of the less dominant bacterial genera were observed (0.0007 ± 0.002 and 0.0009 ± 0.001 vs 0.0011 ± 0.002 and 0.0003 ± 0.0007, for an unknown genus *Gemellaceae* family, *p* = 0.017; and 0.0011 ± 0.0026 and 0.0047 ± 0.0102 vs 0.0015 ± 0.0031 and 0.0011 ± 0.0023, *p* = 0.032 for *Anaerofustis*, percentage relative abundance, V1 and V2, CTRL vs OEP, respectively). Figure 3a shows the change in relative abundance at genus level for *Bifidobacterium, Ruminococcus* and *Lactobacillus* for OEP and CTRL treatments between V1 and V2.

#### Culture-independent targeted quantitative enumeration of faecal bacteria

**Fig. 4:**
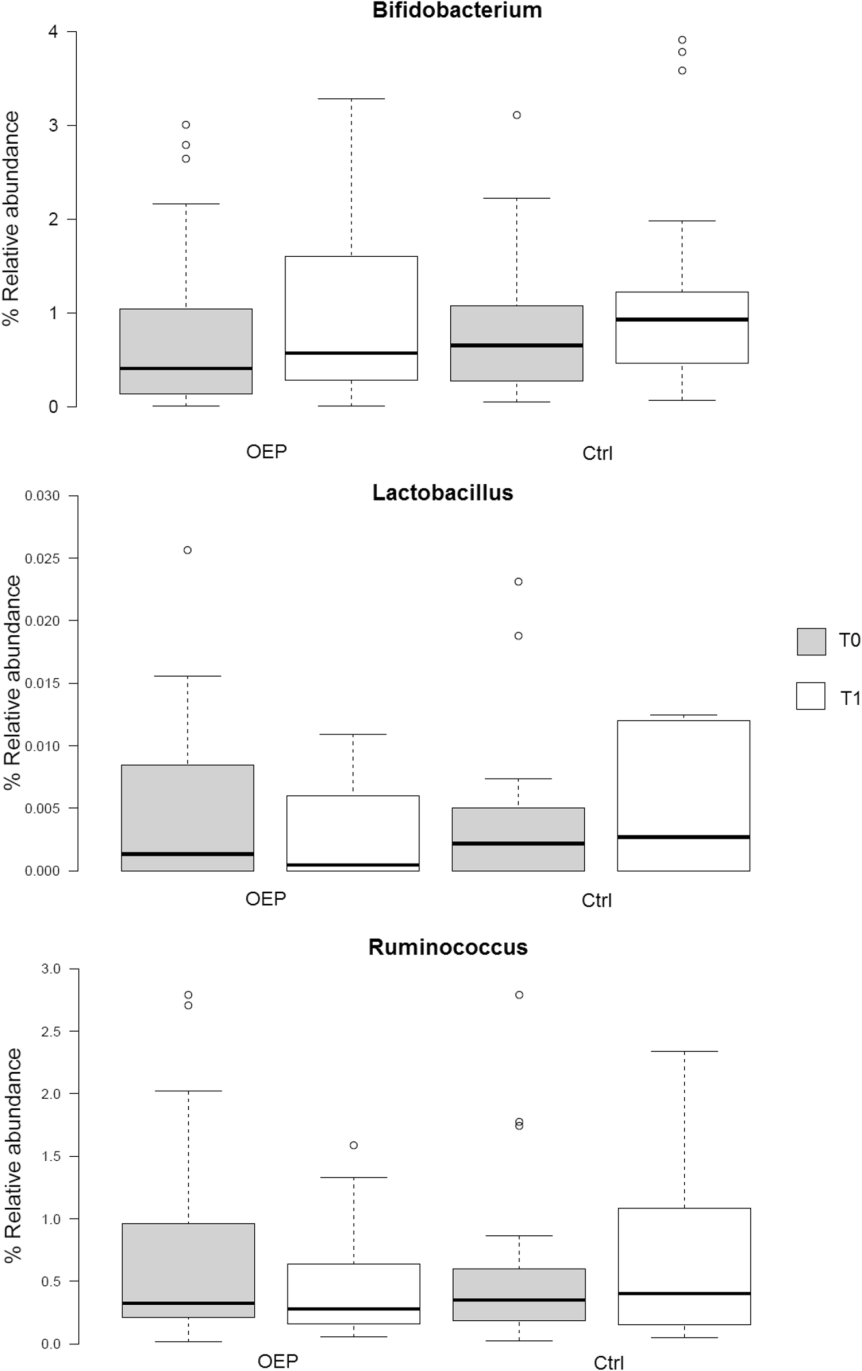
Difference in percentage relative abundance of relevant bacterial genera before (T0) and after (T1) dietary intervention with olive-enriched product (OEP) or control product (Ctrl). *p* = 0.73, *p* = 0.034 and *p* = 0.02, respectively, for *Bifidobacterium, Lactobacillus* and *Ruminococcus* genera, after comparison of the difference T1–T0 between olive-enriched product (OEP) and control product (Ctrl), according to Mann–Whitney *U* test. Center lines show the medians; box limits indicate the 25th and 75th percentiles as determined by R software; whiskers extend 1.5 times the interquartile range from the 25th and 75th percentiles; outliers are represented by dots

No significant differences were observed between treatment OEP and CTRL compared to baseline values (1.85 ± 2.89 and 2.17 ± 3.33 vs 1.39 ± 1.70 and 2.20 ± 3.88) for *Bifidobacterium* spp. (1.07 ± 1.57 and 1.06 ± 1.31 vs 0.53 ± 0.78 and 0.61 ± 1.11) for *Lactobacillus*/*Enterococcus* spp. (0.58 ± 0.97 and 0.54 ± 0.54 vs 0.50 ± 0.79 and 0.59 ± 0.87, for *Ruminococcus obeum* group V1 and V2, OEP vs CTRL, respectively, *p* > 0.05 factorial ANOVA). Figure 4 shows the % of bacteria enumerated with oligonucleotidic probes specific for *Bifidobacterium* spp., *Lactobacillus*/*Enterococcus* spp. and *Ruminococcus* spp.

#### Quantitative PCR

No significant changes in faecal bifidobacteria (6.34 ± 0.95 and 6.62 ± 0.92 vs 6.67 ± 0.74 and 6.72 ± 0.74, V1 and V2, P vs W, respectively, *p* > 0.05, factorial ANOVA) or in total faecal bacteria (10.08 ± 0.04 and 10.08 ± 0.05 vs 10.07 ± 0.05 and 10.07 ± 0.04, V1 and V2, OEP vs CTRL, respectively, *p* > 0.05, factorial ANOVA) were observed after qPCR analysis (data not shown).

### Urinary metabolites quantified by LC–MS

The results of targeted urinary polyphenols are shown in Table 3. Metabolites which were below the detection limit in the majority of samples were excluded from further analysis. Statistical analysis (factorial ANOVA) showed that a number of polyphenol metabolites were significantly higher after OEP treatment compared to CTRL. In particular, 3-3-hydroxyphenyl propanoic acid (*p* = 0.009), 3,4-dihydroxyphenyl acetic acid (*p* < 0.001), hippuric acid (*p* = 0.014), caffeic acid (*p* = 0.003), homovanillic acid (*p* < 0.001), 3-hydroxyphenyl acetic acid (*p* = 0.001), sinapic acid (*p* = 0.002), scopoletin (*p* = 0.001). 2,4-Dihydroxybenzoic acid (*p* < 0.001), 2,5-dihydroxybenzoic acid (*p* = 0.022), 3-(3-hydroxyphenyl) propionic acid (*p* = 0.009), were increased after OEP feeding.

**Table 3:** Urinary polyphenol concentration (µM) quantified by mass spectrometry and normalized according to 24-h urine volume

Treatment time	OEP		Ctrl		*p* value
	T0	T1	T0	T1	
Anthranilic acid (µM)					
Mean	1.30	1.38	1.33	1.46	0.467
SD	0.98	0.94	0.86	1.39	
4-Aminobenzoic acid (µM)					
Mean	0.03	0.02	0.03	0.03	0.173
SD	0.02	0.01	0.01	0.02	
Vanillin (µM)					
Mean	30.63	12.35	38.98	27.56	0.071
SD	66.66	21.52	66.17	60.26	
Acetovanillone (µM)					
Mean	2.66	2.58	3.13	2.60	0.435
SD	2.45	1.47	3.64	1.87	
2,4-DiOH-benzoic acid (µM)					
Mean	0.81	1.09	0.92	0.83	< 0.001
SD	0.34	0.44	0.40	0.36	
3,5-DiOH-benzoic acid (µM)					
Mean	3.01	2.25	3.65	3.11	0.483
SD	1.93	1.64	3.32	2.86	
2,5-DiOH-benzoic acid (µM)					
Mean	2.93	3.26	2.53	2.26	0.022
SD	2.83	2.29	1.28	1.74	
Neochlorogenic acid (µM)					
Mean	0.43	0.35	0.32	0.35	0.149
SD	0.43	0.31	0.22	0.25	
Chlorogenic acid (µM)					
Mean	0.59	0.57	0.55	0.46	0.738
SD	0.44	0.51	0.51	0.41	
Fertaric acid (µM)					
Mean	0.12	0.11	0.11	0.13	0.475
SD	0.23	0.12	0.14	0.18	
*t*-Coutaric acid (µM)					
Mean	0.04	0.03	0.05	0.05	
SD	0.07	0.04	0.08	0.11	0.614
Phloretin (µM)					
Mean	0.04	0.02	0.05	0.03	
SD	0.07	0.02	0.09	0.06	0.214
Phlorizin (µM)					
Mean	0.10	0.05	0.09	0.07	
SD	0.16	0.07	0.21	0.11	0.368
Naringenin (µM)					
Mean	0.07	0.04	0.10	0.08	
SD	0.08	0.03	0.11	0.10	0.709
Phloroglucinol (µM)					
Mean	15.09	15.43	15.68	15.54	
SD	5.49	5.93	6.26	6.26	0.788
4-Hydroxybenzoic acid (µM)					
Mean	6.27	6.17	6.81	5.24	
SD	3.87	4.13	3.06	2.24	0.076
*m*-Coumaric acid (µM)					
Mean	0.67	0.81	0.68	0.60	
SD	0.87	0.79	0.87	0.66	0.341
3-(3-Hydroxyphenyl) propanoic acid (µM)					
Mean	20.65	34.02	16.12	13.83	
SD	18.12	24.58	21.20	18.33	0.009
Vanillic acid (µM)					
Mean	2.80	3.16	2.32	2.25	
SD	2.50	2.19	2.39	1.95	0.499
3,4-Dihydroxyphenyl acetic acid (µM)					
Mean	1.39	22.09	1.68	0.94	
SD	3.04	13.19	3.28	2.11	< 0.001
Hippuric acid (µM)					
Mean	746.62	917.46	789.12	734.80	
SD	277.46	331.38	321.79	313.13	0.014
Caffeic acid (µM)					
Mean	0.45	0.64	0.47	0.44	
SD	0.34	0.28	0.24	0.24	0.003
Homovanillic acid (µM)					
Mean	20.96	54.41	21.63	19.62	
SD	7.36	21.87	8.12	8.02	< 0.001
Isoferulic acid (µM)					
Mean	1.45	1.72	0.59	0.93	
SD	3.88	4.62	0.61	1.76	0.577
*t*-Ferulic acid (µM)					
Mean	2.47	2.35	1.71	3.07	
SD	2.62	2.40	1.17	6.01	0.901
Alpha-hydroxyhippuric acid (µM)					
Mean	0.06	0.05	0.04	0.05	
SD	0.07	0.05	0.05	0.06	0.829
Urolithin A (µM)					
Mean	0.26	0.23	0.49	0.36	
SD	0.40	0.58	1.11	0.86	0.623
3-Hydroxyphenyl acetic acid (µM)					
Mean	34.82	65.11	28.76	29.58	
SD	40.05	43.29	18.77	27.69	0.001
Hydroferulic acid (µM)					
Mean	3.95	5.47	4.87	3.82	
SD	5.20	5.21	5.19	3.71	0.106
Sinapic acid (µM)					
Mean	0.64	1.06	0.78	0.67	
SD	0.51	0.78	0.71	0.69	0.002
Protocatechuic acid (µM)					
Mean	0.55	0.75	0.54	0.50	
SD	0.59	0.39	0.32	0.28	0.001
Scopoletin (µM)					
Mean	0.14	0.29	0.11	0.13	
SD	0.14	0.16	0.12	0.15	0.001
Cryptochlorogenic acid (µM)					
Mean	0.15	0.14	0.12	0.14	
SD	0.20	0.15	0.15	0.19	0.880
Syringic acid (µM)					
Mean	0.48	0.64	0.62	0.59	
SD	0.27	0.49	0.50	0.47	0.083
4-Hydroxyphenyl acetic acid (µM)					
Mean	109.73	125.51	96.81	101.87	
SD	86.90	65.10	40.88	57.97	0.290

Data represent the mean and standard deviation (SD) at the beginning (T0) and at the end (T1) of dietary intervention with olive-enriched product (OEP) or control product (Ctrl), and the relative *p* value after factorial ANOVA with Bonferroni’s correction. Cinnamic acid, caftaric acid, *cis*-piceide, luteolin, hesperidin, catechin, epicatechin, procyanidin B1, procyanidin B2 + B4, procyanidin B3, quercetin-3-Rha, kaempferol-3-Glc, kaempferol-3-rutinoside, dihydrokaempferol, quercetin-3-glucuronide, kaempferol-3-glucuronide, arbutin, *p*-coumaric acid, *o*-coumaric acid, gallic acid, ellagic acid, pyrocatechol, urolithin B, epigallocatechin gallate, epicatechin gallate, quercetin-3-Glc + quercetin-3-gal, isorhamnetin-3-Glc, rutin, salidroside are not shown, since the levels fell below limit of quantification

### Plasma metabolites quantified by LC–MS

The results of targeted quantification of plasma metabolites by LC–MS are shown in Table 4. Metabolites which were below the detection limit in the majority of samples were excluded from further analysis. Statistical analysis (factorial ANOVA) showed that 3,4-dihydroxyphenyl acetic acid (*p* = 0.002) and homovanillic acid (*p* = 0.003) were significantly higher after OEP treatment compared to CTRL. Most of the polyphenol metabolites were present at very low concentrations in plasma compared to urine since the plasma samples were taken in a fasted state.

**Table 4:** Plasma polyphenol concentration (µM) quantified by mass spectrometry

Treatment time	OEP		Ctrl		*p* value
	T0	T1	T0	T1	
Anthranilic acid (µM)					
Mean	0.013	0.018	0.016	0.016	0.709
SD	0.012	0.023	0.015	0.015	
Vanillin (µM)					
Mean	0.092	0.087	0.078	0.065	0.614
SD	0.090	0.058	0.042	0.030	
2,4-DiOH-benzoic acid (µM)					
Mean	0.006	0.008	0.007	0.005	0.891
SD	0.009	0.011	0.013	0.008	
Phlorizin (µM)					
Mean	0.006	0.009	0.005	0.021	0.974
SD	0.008	0.028	0.007	0.080	
Pyrogallol (µM)					
Mean	0.182	0.157	0.238	0.235	0.738
SD	0.319	0.357	0.584	0.465	
4-Hydroxybenzoic acid (µM)					
Mean	0.217	0.046	0.274	0.094	0.798
SD	0.303	0.086	0.301	0.166	
3-(3-Hydroxyphenyl propanoic acid (µM)					
Mean	1.449	1.614	2.286	1.735	0.551
SD	0.784	1.292	3.646	1.098	
Vanillic acid (µM)					
Mean	0.041	0.033	0.041	0.055	0.689
SD	0.028	0.020	0.025	0.099	
3,4-Dihydroxyphenyl acetic acid (µM)					
Mean	0.010	0.651	0.006	0.021	< 0.001
SD	0.032	0.493	0.031	0.062	
Hippuric acid (µM)					
Mean	11.200	14.524	13.034	14.003	0.355
SD	7.438	9.166	10.503	11.230	
Caffeic acid (µM)					
Mean	0.024	0.018	0.013	0.015	0.651
SD	0.045	0.023	0.016	0.018	
Homovanillic acid (µM)					
Mean	0.093	0.217	0.069	0.090	0.003
SD	0.075	0.135	0.061	0.057	
Syringic acid (µM)					
Mean	0.015	0.007	0.012	0.032	0.093
SD	0.021	0.014	0.016	0.109	
4-Hydroxyphenyl acetic acid (µM)					
Mean	2.327	1.449	1.140	1.232	0.525
SD	5.450	1.521	1.131	1.209	0.709

Data represent the mean and standard deviation (SD) at the beginning (T0) and at the end (T1) of dietary intervention with olive-enriched product (OEP) or control product (Ctrl), and the relative *p* value after factorial ANOVA with Bonferroni’s correction

Chlorogenic acid, *cis*-piceide, quercetin-3-Rha, quercetin-3-glucuronide, kaempferol-3-glucuronide, *t*-ferulic acid, ellagic acid, protocatechuic acid, cryptochlorogenic acid, quercetin-3-Glc + quercetin-3-gal, hydroxytyrosol and tyrosol are not shown, since the levels fell below limit of quantification

### Clinical measures of CVD risk and inflammation

Subjects in either group, OEP or CTRL, were matched for age and sex. Little difference was observed in baseline clinical parameters between the groups before dietary intervention. After 8 weeks of treatment with either biscuit, no significant change in CVD or inflammatory makers was observed (Table 2). There was a trend towards reduced oxidized LDL cholesterol in the OEP-treated group, but this was not significant either with respect to time or compared to the control treatment.

### Sex impacted on urinary metabolite profiles

**Fig. 5:**
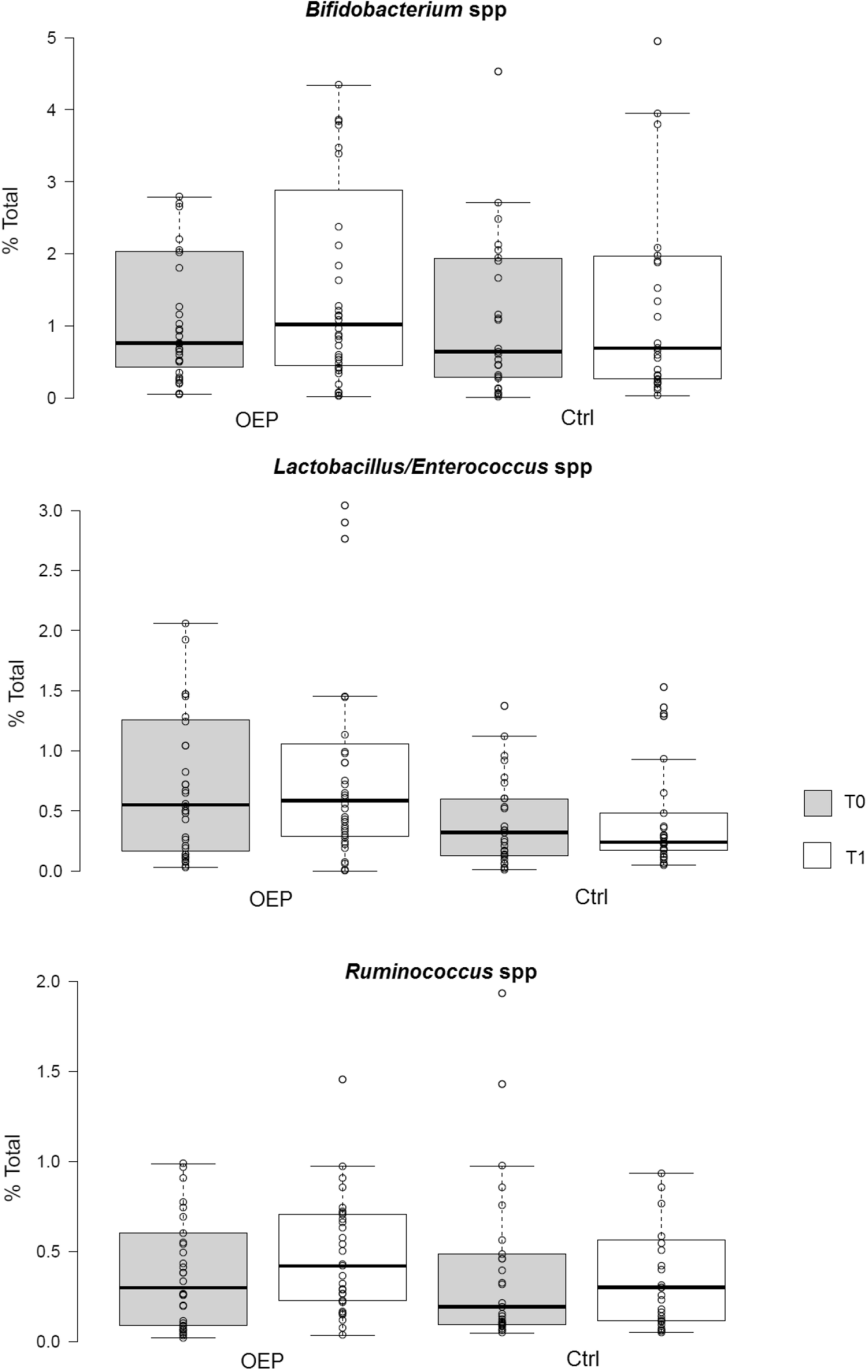
Bacterial populations enumerated by FCM-FISH (% of total bacteria enumerated by SYBR green staining) in faecal samples collected before (T0) and after (T1) dietary intervention with olive-enriched product (OEP) or control product (Ctrl). Center lines show the medians; box limits indicate the 25th and 75th percentiles as determined by R software; whiskers extend 1.5 times the interquartile range from the 25th and 75th percentiles; outliers are represented by dots

**Fig. 6:**
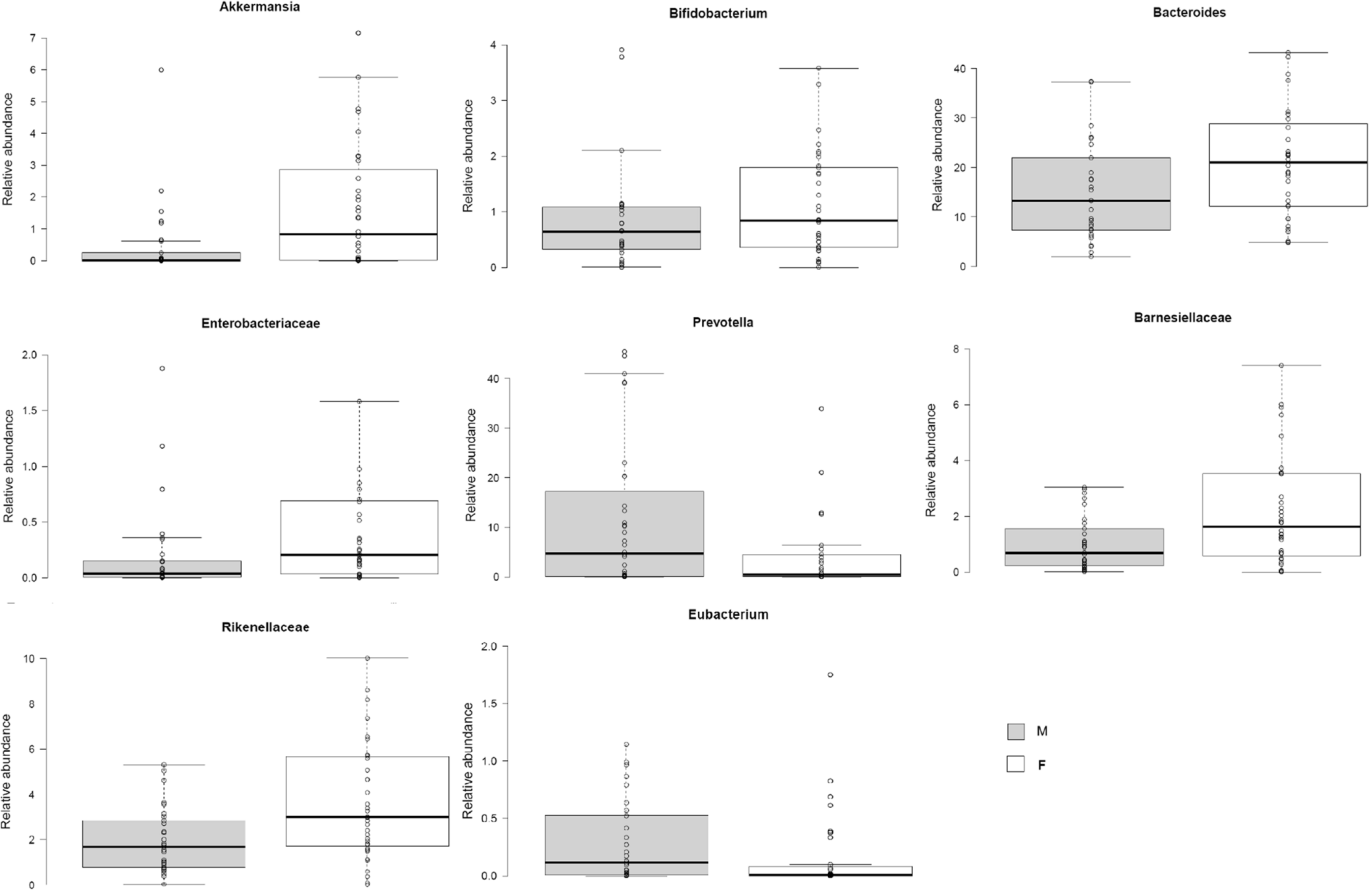
Significant differences between male (M) and female (F) in percentage relative abundance of bacterial genera after dietary intervention with olive-enriched product (OEP), according to Mann–Whitney *U* test. Center lines show the medians; box limits indicate the 25th and 75th percentiles as determined by R software; whiskers extend 15 times the interquartile range from the 25th and 75th percentiles; outliers are represented by dots

Although sex did not appear to influence to response to OEP ingestion in urine or in plasma, differences were observed in the concentrations of small phenolic acids excreted by women compared to men after the OEP treatment. Higher amounts of 3,5-diOH-benzoic acid, *t*-coutaric acid, naringenin, 4-hydroxybenzoic acid, 4-hydroxyphenyl acetic acid were excreted by male subjects compared to female subjects after the OEP treatment (Table 5). Since the gut microbiota is intricately involved in the metabolism of complex polyphenolic compounds and especially in the production of small phenolic acids, we measured whether the gut microbiota of male and female subjects differed after OEP ingestion. Statistically significant differences in the relative abundance of *Akkermansia, Bifidobacterium, Bacteroides, Prevotella, Rikenellaceae, Barnesiellaceae*, and *Enterobacteriaceae* were observed between the faecal microbiota of men and women (Fig. 5). These differences were statistically significant after correction for repeated measures (Mann–Whitney *U* test, 2*1 exact *p* value) (Fig. 6).

**Table 5:** Total urine polyphenols that appeared significantly different between male (M) and female (F) volunteers at the end of intervention (T1) with olive-enriched product (OEP)

Treatment time	OEP		*p* value
	T1		
Gender	M	F	
3,5-DiOH-benzoic acid (µM)			
Mean	2.89	1.80	0.007
SD	1.44	1.65	
*t*-Coutaric acid (µM)			
Mean	0.04	0.02	0.021
SD	0.04	0.04	
Naringenin (µM)			
Mean	0.05	0.03	0.015
SD	0.03	0.03	
4-Hydroxybenzoic acid (µM)			
Mean	6.68	5.81	0.023
SD	2.19	5.12	
4-Hydroxyphenyl acetic acid (µM)			
Mean	153.66	105.41	0.036
SD	80.96	42.55	

Table shows the *p* values after factorial ANOVA with Bonferroni’s correction. Data represent mean and standard deviation (SD) of urinary concentrations (µM), after normalization according to 24-h urine volume

## Discussion

The primary objective of this study was to measure the impact of an olive pomace-enriched product (OEP) on the composition and metabolic output of the human gut microbiota. Considering the accepted physiological relevance of olive polyphenols, their apparent ability to protect LDL cholesterol particles from oxidative damage, and the fact that the gut microbiota appears to be intimately related to their metabolism in vivo, we measured changes in key olive-derived polyphenols, including tyrosol and HT, and their derived catabolites using a quantitative LC–MS-based strategy. The OEP biscuits did not have a major impact on the composition of the gut microbiota, but did induce subtle changes in relative abundances of certain bacteria. Significant differences in relative abundance of *Lactobacillus, Ruminococcus, Gemellaceae* and *Anaerofustis* were observed between treatments using community level 16S rRNA profiling. More quantitative analysis using flow cytometry-coupled fluorescent in situ hybridization did not confirm statistically significance for bifidobacteria, lactobacilli or the *Ruminococcus obeum*-like bacteria. However, a trend was apparent, consistent between 16S rRNA gene sequencing, the probe-based FISH and qPCR, showing a small increase in bifidobacteria.

In terms of metabolic output, LC–MS-based targeted metabolomics confirmed that ingestion of the OEP biscuits resulted in a significant increase in urinary excretion of small phenolic acids derived from the metabolism of olive polyphenols. These small phenolic acids derive from the combined activities of human phase I and II biotransformation and the action of the gut microbiota. OEP ingestion resulted in a significant increase in excretion of homovanillic acid, 3,4-dihydroxyphenyl acetic acid, scopoletin, protocatechuic acid, sinapic acid, 3-hydroxyphenyl acetic acid, isoferulic acid, caffeic acid, hippuric acid, 3,3-hydroxyphenyl acetic acid, 2,5-dihydroxybenzoic acid and 2,4-dihydroxybenzoic acid. Many of these compounds derive from the breakdown pathways of the tyrosol group enriched in olives and/or the hippuric acid pathway, a pathway common to many classes of polyphenols. Both involve steps mediated by the gut microbiota and these catabolites and similar small phenolic acids have been reported to be excreted following ingestion of olive or olive fractions in previous studies [20–22, 36, 37]. Few studies have reported the profile of metabolites present in fasted blood samples after chronic ingestion of olive pomace. Here we found that ingestion of the OEP biscuits for 8 weeks resulted in a significantly higher level of tyrosol metabolites, homovanillic acid and 3,4-dihydroxyphenyl acetic acid (DOPAC). Homovanillic acid increased more then twofold in the OEP-treated group and was also about double the concentration after the CTRL treatment. For DOPAC, OEP induced more than tenfold increase in fasted blood concentrations, and a similar difference in magnitude was observed compared to the control group. As well as being associated with the protection of LDL cholesterol particles from oxidative damage, both homovanillic acid and DOPAC have been reported to transiently associate with LDL cholesterol particles in blood thereby mediating their antioxidative effect. While most studies report these molecules to be relatively rapidly cleared from the blood [38], our study is one of the very few to record high levels of these antioxidants in fasted blood samples, many hours after ingestion of the olive-enriched food. This is an important observation for the possible functional activities of the OEP biscuit since persistence of these tyrosol-derived metabolites in blood, or indeed within the intestine, could prime antioxidant defences and/or absorption of oxidized LDL cholesterol upon fat meal challenge. However, our current study was not designed to measure the effect of olive polyphenols on oxidized LDL cholesterol levels and therefore, any potential beneficial effect of the OEP biscuit awaits confirmation in an acute, postprandial study. Similarly, homovanillic acid and DOPAC are both involved in the dopamine pathway and have been shown to mediate other physiological effects including ameliorating age-related decline in muscle function [39] and brain function [40]. In addition, DOPAC has been shown to impact on the inflammatory response of immune cells to lipopolysaccharide or endotoxin, an inflammatory microbially derived signal associated with increased risk of metabolic disease [41]. However, any ability of the OEP biscuits to mediate such health effects awaits specifically and appropriately designed human studies.

In this current study, we also measured the ability of the OEP biscuit to modulate blood lipid profiles. Previous studies with whole plant foods or oat-derived beta-glucan in particular, have shown significant and clinically meaningful reductions in cholesterol upon ingestion [42]. However, the mechanisms by which these foods mediate their cholesterol lowering effects are still very much unclear, with different mechanisms suggesting involving phytosterols, gel-forming and cholesterol binding activities, modified bile acid profiles and/or prebiotic type modulation of the gut microbiota [43, 44]. Our OEP, not containing the pit pulp of the olive, did not contain large amounts of plant phytosterols, and only had minor impact on microbiota composition. Although in this case, the olive pomace did not change blood lipid profiles, further studies, possibly with larger sample size be warranted, especially since the ability of olive and olive extracts in general to modulate blood lipid profiles remains to be convincingly established as per the EFSA statement on olive polyphenol extract health claims [18].

The quantities of small phenolic acids in urine differed between men and women upon OEP ingestion. Men excreted significantly more 3,5-diOH-benzoic acid, *t*-coutaric acid, naringenin, 4-hydroxybenzoic acid, 4-hydroxyphenyl acetic acid than women. A sex bias in polyphenol metabolism has been reported previously. Zamora-Ros et al. [45] analysing the EPIC cohort study reported a significant sex bias in excretion of dietary polyphenols. Moreover, the sex differences in dietary polyphenol metabolism also appears to be reflected in the concentration and profiles of polyphenols or their derivatives in different tissues and organs, as shown for grape seed flavanols in rats [46]. Such sex effects could have important implications for the biological activity of these compounds, especially since a sex effect has also been reported in response to dietary interventions measuring physiological change and/or reduced risk of chronic disease upon intervention with polyphenol-rich foods [47]. The contribution of the intestinal microbiota to any sex-specific “metabotype” in terms of polyphenol metabolism remains very much unexplored. In our study, faecal samples collected from men and women post-OEP ingestion differed significantly in the relative abundance of *Akkermansia, Bifidobacterium, Bacteroides, Prevotella*, Rikenellaceae, Barnesiellaceae, and Enterobacteriaceae. Some of these bacteria are linked to host physiology and protection from metabolic and cardiovascular disease (*Akkermansia* and *Bifidobacterium* in particular), but also *Bacteroides* and *Prevotella* in relation to obesity and traditional dietary paradigms [48–50]. Similarly, the enterobacteria, which includes many intestinal pathogens, appear particularly susceptible to the antibacterial activities of polyphenols [51]. The role of the human gut microbiota in determining the profile and quantities of different polyphenol breakdown products, their bioavailability, bioactivity and nutri-kinetics may constitute and important new compounding factor to be taken into consideration when designing human dietary interventions where polyphenols are considered mediators of physiological effect. Further data from similar human studies are required to confirm the involvement of the gut microbiota in gender-specific polyphenol metabolism and possible implications sex-specific response to diet.

In conclusion, ingestion olive pomace extract-enriched biscuits mediated small changes within the composition of the gut microbiota. Delivering 17.1 ± 4.01 mg/100 g HT and its derivatives, the OEP biscuits induced a significant increase in excretion of small phenolic acids in urine, indicative of up-regulation of microbial polyphenol biotransformation in the intestine. Quantities of some small phenolic acids differed in urine of men and women, as did relative abundances of important members of the gut microbiota. OEP also led to a significant increase in homovanillic acid and DOPAC in fasted plasma samples, indicating related clearance of these compounds from the blood or extended release and uptake from the intestine. In either case, higher levels of these biologically active compounds mediated by OEP ingestion warrant further investigation in acute or post-prandial studies specifically targeting LDL cholesterol oxidation and cognitive function.
